# Adapting to survive: How *Candida* overcomes host-imposed constraints during human colonization

**DOI:** 10.1371/journal.ppat.1008478

**Published:** 2020-05-21

**Authors:** Rosana Alves, Cláudia Barata-Antunes, Margarida Casal, Alistair J. P. Brown, Patrick Van Dijck, Sandra Paiva

**Affiliations:** 1 Centre of Molecular and Environmental Biology, University of Minho, Campus de Gualtar, Braga, Portugal; 2 Institute of Science and Innovation for Bio-Sustainability (IB-S) University of Minho, Campus de Gualtar, Braga, Portugal; 3 MRC Centre for Medical Mycology, University of Exeter, Exeter, United Kingdom; 4 VIB-KU Leuven Center for Microbiology, Flanders, Belgium; 5 Laboratory of Molecular Cell Biology, Institute of Botany and Microbiology, KU Leuven, Leuven, Belgium; University of California Los Angeles David Geffen School of Medicine, UNITED STATES

## Abstract

Successful human colonizers such as *Candida* pathogens have evolved distinct strategies to survive and proliferate within the human host. These include sophisticated mechanisms to evade immune surveillance and adapt to constantly changing host microenvironments where nutrient limitation, pH fluctuations, oxygen deprivation, changes in temperature, or exposure to oxidative, nitrosative, and cationic stresses may occur. Here, we review the current knowledge and recent findings highlighting the remarkable ability of medically important *Candida* species to overcome a broad range of host-imposed constraints and how this directly affects their physiology and pathogenicity. We also consider the impact of these adaptation mechanisms on immune recognition, biofilm formation, and antifungal drug resistance, as these pathogens often exploit specific host constraints to establish a successful infection. Recent studies of adaptive responses to physiological niches have improved our understanding of the mechanisms established by fungal pathogens to evade the immune system and colonize the host, which may facilitate the design of innovative diagnostic tests and therapeutic approaches for *Candida* infections.

## Introduction

The human body is home to a large number of microbes that play essential roles in maintaining human health. However, under particular host-compromising conditions, they can shift from harmless commensals to opportunistic pathogens to cause inflammation and disease. Fungal communities, which can include *Candida* species, constitute an integral part of the human microbiota that, under normal conditions, asymptomatically colonize several niches, including the skin, oral cavity, gastrointestinal, and urogenital tracts [1–3]. The remarkable ability to alternate between local current microenvironments within internal host niches such as blood or tissues is often linked with their pathogenic potential. Therefore, environmental changes promoted either by alterations in host microbiota or the host immune system may allow these microorganisms to overgrow, cross the epithelial barriers, and cause severe, life-threatening infections [4].

Among the *Candida* species that trigger human disease, *Candida albicans*, *C*. *glabrata*, *C*. *parapsilosis*, *C*. *tropicallis*, and *C*. *krusei* are the most common [4–6]. Yet, other emerging species, including *C*. *auris*, *C*. *guilliermondii*, *C*. *lusitaniae*, and *C*. *metapsilosis*, are of particular concern because they are rapidly spreading worldwide, with several reported outbreaks [5,7,8]. Moreover, *Candida* infections are difficult to diagnose, commonly resulting in delayed antifungal treatments that have been associated with hospital mortality [9]. The antifungal drugs available to eradicate these fungal pathogens are also limited and often ineffective, mainly because of the intrinsic multidrug resistance of certain *Candida* species and their ability to form biofilms on implanted medical devices [10–12]. Considering that each species presents its own distinctive features in relation to invasive potential, morphogenesis, antifungal susceptibility, and biofilm formation, studies focusing on the adaptation to different hosts and environmental factors have the potential to reveal novel molecular players of virulence pathways.

**Fig 1:**
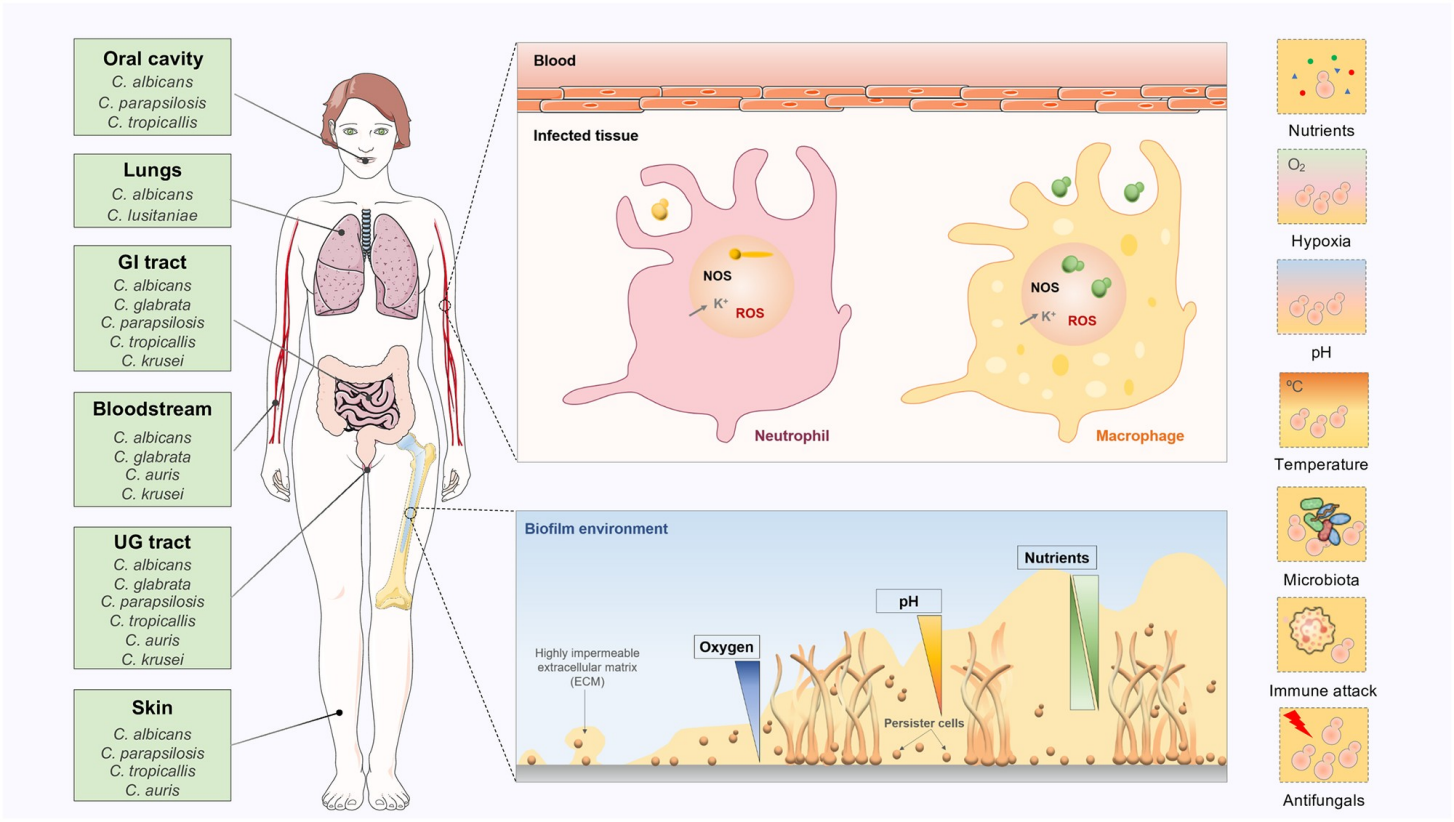
*Candida* biogeography and the different host-imposed constraints during human colonization. The most frequently isolated *Candida* species are listed according to their principal habitat in the human body (oral cavity, lungs, gastrointestinal tract, bloodstream, urogenital tract, and skin). The different host-imposed constraints are highlighted for several microenvironments where *Candida* thrives in the human body, including inside phagocytic cells or biofilms. Key references: *C*. *albicans* [2,3], *C*. *glabrata* [3], *C*. *parapsilosis* [2], *C*. *tropicalis* [2], *C*. *lusitaniae* [13], and *C*. *krusei* [6]. ECM, extracellular matrix; NOS, nitric oxide species; ROS, reactive oxygen species; UG, urogenital.

Here, we provide an overview of established and emerging strategies used by *Candida* to adapt to common environmental challenges faced by these fungi during immune evasion and human colonization (Fig 1). As we review major host-imposed constraints, we highlight the central regulatory circuits required for fungal adaptation to these challenges. We also discuss the impact of such physiological reprogramming on key aspects of *Candida* pathogenicity, with a particular emphasis on immune evasion, biofilm formation, and antifungal drug resistance. We propose that the genetic circuits governing *Candida* adaptation to human niches can be exploited in search of new antifungal targets and diagnosis improvement.

## *Candida* within the human host

The human host contains a variety of environmental niches in which *Candida* species can thrive. Adaptation to these sites requires rapid and coordinated changes in *Candida* metabolism and physiology in order to avoid or escape immune surveillance and to counteract several host-imposed constraints (for example, nutrient limitation, oxygen deprivation, pH fluctuations, changes in temperature, or oxidative, nitrosative, and cationic stresses). Moreover, *Candida* species interact with other microbial residents, establishing either cooperative or antagonistic relationships, which may affect their growth and influence the outcome of an infection.

Depending on the local environmental cues, some *Candida* species may exhibit different cellular morphologies. These include budding forms, which have been associated with commensalism, and the filamentous forms hyphae and pseudohyphae, often related with invasive and disseminated disease [14,15]. However, these cell types were found in infected tissues, suggesting they all promote pathogenicity. *C*. *albicans* has also the ability to switch into more functionally and genotypically distinct cell types, which may present improved fitness in specific host niches [15]. In particular, “white” yeast cells can switch to mating specialized “opaque” cells, and a subset of these can also transit into a third, “gray” morphology [16]. An additional distinctive group of cells, known as GUT (gastrointestinally induced transition), seems to display enhanced fitness in the gastrointestinal tract when compared with other cell types [17]. The morphogenic transitions depend on a highly dynamic cell wall that acts as an environmental barrier, and it is essential for host–pathogen interactions. The core skeleton of the cell wall is composed of the polysaccharide β-1,3-glucan, covalently linked to β-1,6-glucan and chitin. The outer layer contains glycosylated mannoproteins cross-linked to β-1,6-glucans. The relative amount of each component fluctuates between morphologies and in response to external challenges, impacting immune responses [18,19].

## Nutrient availability and *Candida* metabolic flexibility

**Fig 2:**
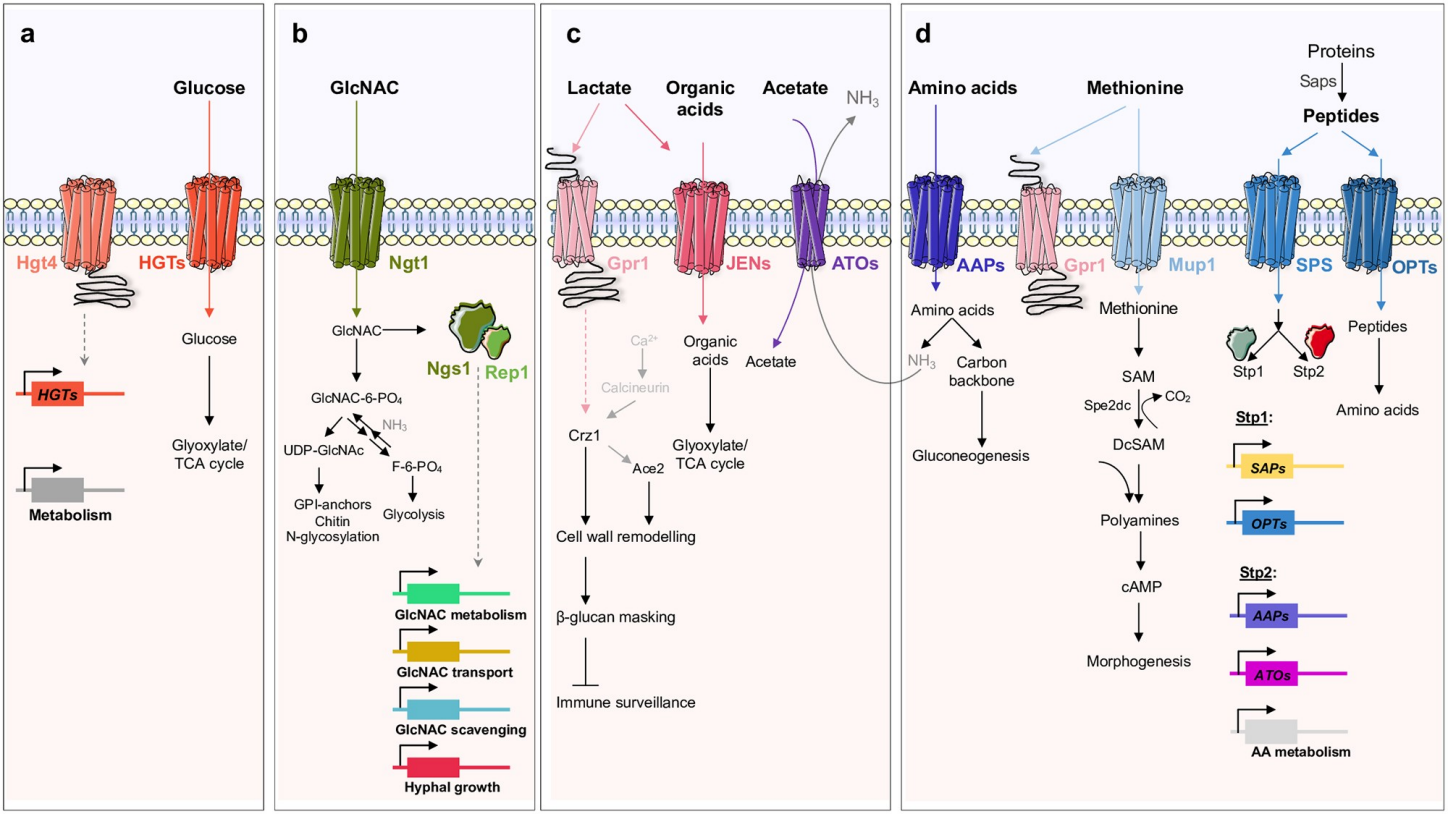
Schematic representation of the main sensing, transport, and transduction systems for the utilization of different host nutrients in *Candida* species. (a) In *C*. *albicans*, glucose is sensed by Hgt4, generating an intracellular signal that induces the expression of HGTs and other metabolic genes. (b) In *C*. *albicans* and *C*. *tropicalis*, the uptake of GlcNAc occurs through the Ngt1 transporter. (c) The uptake of carboxylic acids is facilitated by the Jen (in *C*. *albicans*) and Ato transporters (in *C*. *albicans* and *C*. *glabrata*). In *C*. *albicans*, Gpr1 is reported to be a lactate and methionine sensor. In the presence of lactate, Gpr1 is thought to activate Crz1 in a calcineurin-independent manner and, together with Ace2, regulates a polygenic response that leads to β-glucan masking. (d) Peptides and amino acids are sensed by the SPS complex, which induces the expression of Opts, Aaps, and Ato transporters, as well as SAPs and amino acid catabolic genes. Intracellular ammonia resulting from the catabolism of GlcNAc or amino acids is exported via Ato transporters. In the presence of methionine, and in low glucose conditions, the methionine-induced morphogenesis is activated via Gpr1 sensor and Mup1 transporter. AA, amino acid; Aap, amino acid permease; ATP, adenosine triphosphate; cAMP, cyclic adenosine monophosphate; DcSAM, decarboxylated S-adenosylmethionine; GlcNAc, N-acetylglucosamine; GPI, glycosylphosphatidylinositol; HGT, hexose transporter; Opt, oligopeptide transporter; SAM, S-adenosylmethionine; SAP, secretory aspartyl proteinase; SPS, Ssy1-Ptr3-SSy5; Sp2DC, Sp2 decarboxylase; TCA, tricarboxylic acid cycle; UDP, uridine diphosphate.

Of the many challenges pathogens face in the human host, possibly none is more important than nutrient availability because cells must assimilate nutrients in order to thrive. These might include sugars, carboxylic acids, peptides, amino acids, lipids, or phospholipids. The assimilation of glucose, lactose, and galactose is mediated via hexose transporters (HGTs), providing major sources of energy and carbon (Fig 2a). The well-studied yeast model *Saccharomyces cerevisiae*, which is relatively closely related to some *Candida* species, uses glucose as a preferred carbon source and only switches to nonfermentable nutrients when glucose becomes depleted [20]. This hierarchical utilization requires highly evolved networks integrating several signaling pathways in order to repress the assimilation of alternative carbon sources [21–24]. This is partly achieved by the ubiquitination of key gluconeogenic and glyoxylate cycle enzymes following the exposure to glucose [25]. Notably, these enzymes appear to lack ubiquitination sites in *C*. *albicans*, *C*. *glabrata*, *C*. *parapsilosis*, and *C*. *tropicalis*, and consequently, they are not subjected to glucose-induced degradation [26,27]. The evolutionary rewiring of key metabolic ubiquitination targets has been suggested to increase the ability of *C*. *albicans* to colonize and cause infection in the mammalian host because, unlike *S*. *cerevisiae*, this yeast is able to assimilate sugars and alternative carbon sources simultaneously [26–28]. The availability of glucose is thought to enhance *C*. *albicans* virulence owing to the fact that this sugar has been reported to induce hyphal morphogenesis at low physiological concentrations [29–31] and promote antifungal resistance [32,33]. Moreover, rapid glucose metabolism by *C*. *albicans* seems to be important during infection because immune cells, specifically macrophages, rely on glucose for survival [34]. This limitation is exploited by *C*. *albicans*, which elicits rapid macrophage death by depleting the available glucose [34].

In glucose-limiting conditions, other alternative carbon sources, such as N-acetylglucosamine (GlcNAc) and carboxylic acids, are thought to play a critical role to sustain *Candida* growth. When infecting tissues and organs, *Candida* up-regulates several pathways involved in the utilization of alternative carbon sources, such as gluconeogenesis, the glyoxylate cycle, and fatty acid β-oxidation, suggesting that glucose levels may not be sufficient to satisfy the energetic requirements of the cells [28,35–37]. In *C*. *albicans* and *C*. *tropicalis*, GlcNAc, a monosaccharide produced mainly by bacteria in the gastrointestinal tract, enters the cell through the Ngt1 transporter, and is then sensed by the transcription factors, Ngs1 and Rep1, which control the expression of genes involved in the uptake and catabolism of GlcNAc [38–40] (Fig 2b). Depending on the metabolic state of the cells, GlcNAc can either be converted to uridine diphosphate-N-acetylglucosamine (UDP-GlcNAc) or to fructose-6-phosphate, which then enters the glycolytic pathway (Fig 2b). In *C*. *albicans*, GlcNAc can also be used as a signal to induce the expression of several virulence genes involved in white-opaque switching [41], hyphal morphogenesis [38–40,42], and cell death [43]. Additionally, GlcNAc metabolism seems to sustain *Candida* survival when growing inside phagocytic cells. The export of intracellular ammonia, derived from GlcNAc catabolism, has been reported to promote the alkalization of the phagosome, enabling cells to survive and escape from the acidic environment of the phagolysosome [44]. This mechanism is dependent on the transport of GlcNAc and subsequent catabolism through Hxk1, Nag1, and Dac1 enzymes [44]. Hence, mutants lacking the Ngt1 transporter or GlcNAc catabolic enzymes are defective in neutralizing the phagosome [44]. The ability to manipulate ambient pH is reported for all species of the CTG clade, a phylogenetic group that translates the CUG codon into serine instead of leucine [45]. This is in contrast to what is found for the distantly related *C*. *glabrata*, whose genome does not appear to encode homologs of GlcNAc transporters or catabolic enzymes [44].

*C*. *albicans* can also raise the extracellular pH by metabolizing carboxylic acids [46]. This phenomenon is physiologically and genetically distinct from the GlcNAc-driven mechanism, as the metabolism of carboxylic acids, when used as the sole carbon source, does not generate ammonia or promote hyphal morphogenesis [44,46]. Physiologically relevant carboxylic acids such as lactate, acetate, succinate, butyrate, and propionate are produced either by host cells or host microbiota [47–49]. Lactate and acetate are particularly abundant in the gut and in vaginal secretions [47,50] but also inside phagocytic cells [51,52]. In *C*. *albicans*, the uptake of lactate is mediated by Jen transporters [51,53], while Ato transporters are potentially involved in the transport of acetate in both *C*. *albicans* and *C*. *glabrata* [52,54] (Fig 2c). These two transporter families are strongly induced after phagocytosis [51,52], and they modulate biofilm formation and resistance to antifungal drugs in both *C*. *albicans* and *C*. *glabrata* [54–56]. In particular, exposure to lactate has been shown to trigger the masking of β-glucan, a major pathogen-associated molecular pattern (PAMP), in several *Candida* species [57]. This affects the visibility of these pathogens to host immune defenses, which correlates well with the observed decrease in *C*. *albicans* uptake by macrophages and reduced phagocytic recruitment [57,58]. The β-glucan masking phenotype has been proposed to be dependent on Gpr1 and the transcription factor Crz1 [57]. These proteins control the expression of genes associated with the organization of the cell wall, ultimately contributing to the masking effect [57,59]. Therefore, the concomitant exposure of *Candida* cells to different carboxylic acids potentiates immune evasion and consequently *Candida* persistence.

The uptake of nitrogen is also critical for *Candida* survival. Different in vivo studies have demonstrated that genes involved in amino acid uptake and catabolism are strongly up-regulated in *C*. *albicans*, especially when phagocytosed by neutrophils and macrophages [36,60–62]. Indeed, several *C*. *albicans* and *C*. *glabrata* amino acid auxotrophic strains retain full virulence in mice, suggesting that these nutrients are readily available during infection [63–65]. Proteolytic enzymes, namely secretory aspartyl proteinases (SAPs), are of particular importance because they allow *Candida* to efficiently degrade the complement proteins and host connective tissues [66]. Once available, extracellular amino acids are then sensed by the SPS complex (composed of Ssy1, Ptr3, and Ssy5), which in turn activates the transcription factors, Stp1 and Stp2 (Fig 2d). While Stp1 controls the expression of extracellular proteases and peptide transporters, Stp2 regulates amino acid permeases, Ato transporters, and catabolic enzymes [67,68] (Fig 2d). Along with GlcNAc and carboxylic acids, the catabolism of amino acids represents a third independent mechanism by which *Candida* rapidly neutralizes acidic microenvironments [52,69]. Previous studies reported that *C*. *albicans* mutants lacking *STP2* or *ATO* genes release less ammonia than wild-type controls, failing to efficiently neutralize the acidic phagosome and undergo hyphal morphogenesis, which consequently affects their ability to escape phagocytic cells [52,70]. Recent data, however, suggest that the phagosomal membrane is highly permeable to ammonia, and the observed alkalization is rather a direct consequence of proton leakage induced by hyphal growth [71,72]. The transport of methionine via the high-affinity permease Mup1 and its subsequent metabolism have been also shown to induce morphogenesis in a process that is dependent on Gpr1 and the cAMP-PKA (cyclic Adenosine Monophosphate-Protein Kinase A) signaling cascade [73,74]. The methionine-induced morphogenesis pathway triggers the activation of adenylate cyclase by the production of increased levels of polyamines such as spermine and spermidine. These compounds are generated by the intracellular conversion of methionine into S-adenosylmethionine (SAM) and its decarboxylation by Spe2, which donates aminopropyl groups for polyamine synthesis [73] (Fig 2d).

## Environmental pH fluctuations shape *Candida* physiology and pathogenicity

**Fig 3:**
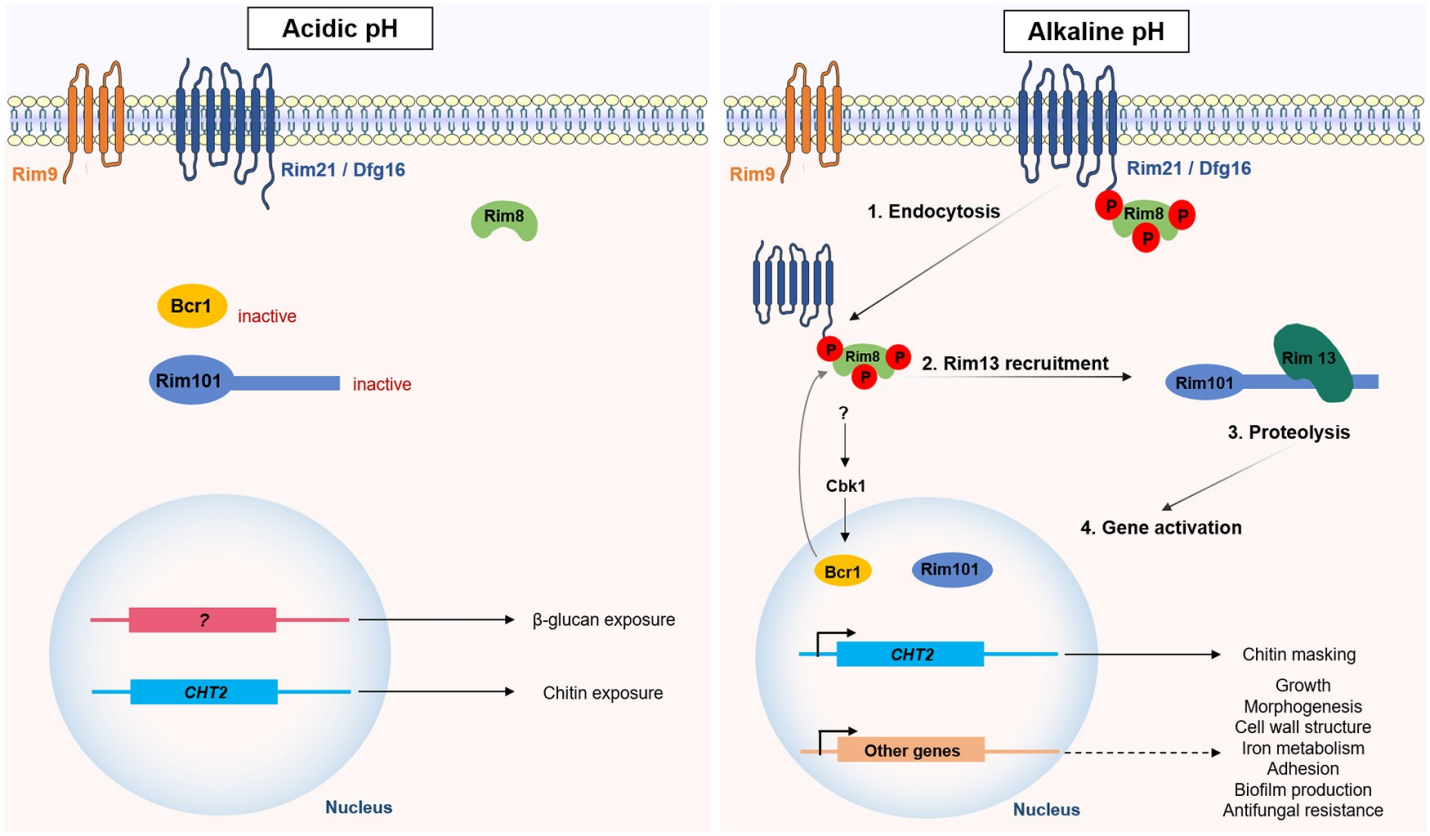
*Candida* adaptation to pH fluctuations. In *Candida* species, pH adaptation is mediated by the Rim pathway. Under acidic pH, the exposure of both chitin and β-glucan is enhanced and facilitates their recognition by the host innate immune system. Chitin exposure is promoted by the repression of both Rim101 and Bcr1, resulting in reduced expression of *CHT2*. β-glucan exposure is regulated by a noncanonical signaling pathway. Under alkaline pH, Rim8 is hyperphosphorylated, a signal that induces the endocytosis of the Rim complex and the recruitment of Rim13. The C-terminal proteolysis of Rim101 by Rim13 activates it and promotes the expression of target genes, including *CHT2*.

Changes in ambient pH represent an additional stress that *Candida* and other pathogens face in the human host. While the pH of human blood and tissues is slightly alkaline (pH 7.4), the pH of the oral cavity and the gastrointestinal and genitourinary tracts is acidic (2 < pH < 6). Adaptation to differing ambient pHs is critical for survival and growth in these niches. In fungi, including *Candida* species, pH signaling is mediated by the Rim pathway [75]. In *C*. *albicans*, the external pH is sensed by Rim21/Dfg16, Rim9, and an arrestin-like protein Rim8. Under alkaline pH, Rim8 is hyperphosphorylated, a signal that triggers the endocytosis of the plasma membrane complex and the recruitment of the signaling protease Rim13. This protease then cleaves the C-terminal inhibitory domain of Rim101, resulting in its activation. The activation of Rim101 promotes the expression of target genes involved in morphogenesis [76–79], growth [80], cell-wall remodeling [80], iron metabolism [81,82], adhesion [80], biofilm formation, and antifungal tolerance [75,83,84] (Fig 3).

On the other hand, the adaptation of *C*. *albicans* to acidic environments drives cell-wall remodeling by enhancing the exposure of two key fungal PAMPs (chitin and β-glucan) at the cell surface [85]. While pH-dependent β-glucan exposure is regulated by a noncanonical signaling pathway, the remodeling of chitin is coordinated by several transcription factors, including Rim101, Bcr1, and Efg1 (Fig 3) [85,86]. The exposure of β-glucan at the cell surface hyperactivates the immune system largely through the recognition of the immunostimulatory β-glucan by Dectin-1, which enhances the recruitment of neutrophils and macrophages to the site of the infection [85]. This pH-dependent β-glucan exposure was also observed in *C*. *dubliniensis* and *C*. *tropicalis*, but not in *C*. *auris* or *C*. *glabrata* [85,86]. Surprisingly, adaptation to acidic environments induces β-glucan masking in *C*. *krusei*, suggesting that the outputs of pH-dependent signal transduction differ between these *Candida* species [85]. Additionally, the pH-dependent reorganization of the cell wall fluctuates over time in *C*. *albicans*, with β-glucan and chitin being masked after an initial period of exposure [86]. While the subsequent β-glucan masking is mediated by farnesol, this quorum-sensing molecule does not trigger the chitin cloaking [86]. These temporal fluctuations suggest dynamic cell-wall responses to environmental pH. Moreover, the early PAMP exposure appears to govern the outcome of the infection because subsequent remasking on the cell wall does not compensate for the initial induction of strong proinflammatory responses [86].

## Adaptation to oxygen-limiting niches is critical for *Candida* virulence

Oxygen levels inside the human host can vary greatly. While some niches are rich in oxygen, such as exposed skin or oral mucosa, others are anoxic or hypoxic, including the gastrointestinal tract [87]. Consequently, *Candida* cells must adapt to low-oxygen environments, particularly when colonizing the human gut, developing lesions or growing in biofilms [87,88]. Analyses of gene expression profiles of *C*. *albicans* cells shifted from normoxia to hypoxic growth conditions revealed the induction of several pathways, including glycolytic gene expression via Tye7 [89–91], fatty acid metabolism [92,93], heme biosynthesis and iron metabolism [89,92,94], cell-wall structure [89,92,94], and sterol biosynthesis via Upc2 [95,96]. In contrast, genes involved in the oxidative respiration were repressed [89,92,94]. Additionally, the Sit4 phosphatase, the Ccr4 mRNA deacetylase, and the Sko1 transcription factor have been identified as potential regulators of an early hypoxic response (10–20 min) [91,94].

**Fig 4:**
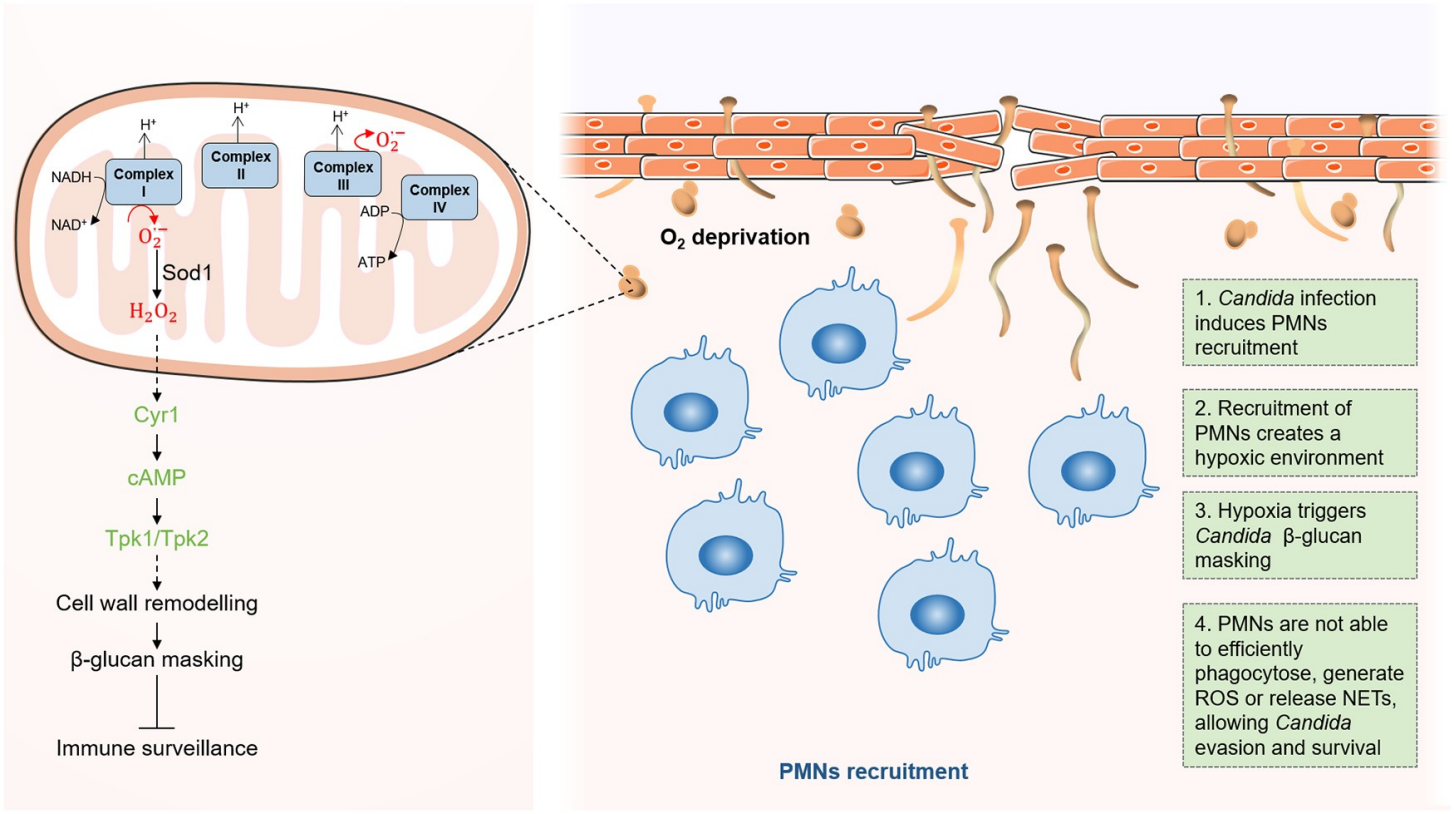
*Candida* adaptation to hypoxic host niches. During *C*. *albicans* infections, the recruitment of PMNs creates an hypoxic environment [88]. In the fungus, this oxygen limitation triggers increased formation of ROS, such as superoxide (O_2_^•−^), from the electron transport chain [98,99]. Superoxide is then converted into diffusible hydrogen peroxide (H_2_O_2_) by the action of Sod1. H_2_O_2_ has been proposed to activate adenylyl cyclase (Cyr1) and cAMP-PKA (Tpk1/2) signaling, which in turn triggers cell-wall remodeling and β-glucan masking [97]. This β-glucan masking allows the fungus to evade phagocytosis by the PMNs [88]. cAMP, cyclic Adenosine Monophosphate; NET, neutrophil extracellular trap; PKA, Protein Kinase A; PMN, polymorphonuclear leukocyte; ROS, reactive oxygen species; Sod1, superoxide dismutase 1.

Besides affecting the cellular metabolism and energy homeostasis, adaptation to hypoxia induces hyphal growth in *C*. *albicans* [94] and promotes immune evasion by triggering β-glucan masking at the cell surface [97]. β-glucan masking leads to reduced phagocytosis and attenuates local immune responses [97]. In contrast to lactate-induced β-glucan masking, hypoxia-induced masking does not depend on Gpr1 and Crz1. Instead, hypoxia-induced masking is mediated by mitochondrial and cAMP-PKA signaling [57,97]. Hypoxia induces the generation of mitochondrial superoxide [98,99], which is rapidly converted into diffusible hydrogen peroxide by superoxide dismutase 1 (Fig 4). Hydrogen peroxide has been proposed to somehow activate the cAMP-PKA pathway, which, in turn, triggers cell-wall remodeling and β-glucan masking [97]. However, the mechanism by which β-glucan masking is achieved at the cell surface remains unclear.

Hypoxia-induced β-glucan masking has been observed for some other pathogenic *Candida* species, namely *C*. *tropicalis* and *C*. *krusei*, but not in *C*. *glabrata*, *C*. *guilliermondi*, or *C*. *parapsilosis* [97]. Therefore, during their evolution, hypoxic signaling has become integrated with PAMP masking only in some *Candida* pathogens. The adaptation to hypoxic environments enhances the ability of these *Candida* species to colonize the host. For example, it was shown that the recruitment of polymorphonuclear leukocytes (PMNs) to sites of *C*. *albicans* infection in mice was the main cause of hypoxia [88] (Fig 4). However, because of the hypoxia-induced β-glucan masking by *C*. *albicans* cells, these PMNs are not able to efficiently phagocytose the fungus, generate reactive oxygen species (ROS), or release extracellular DNA traps, allowing *C*. *albicans* to survive. Continued exposure to hypoxia leads to accumulation of lactate, prolonging the masking effect. Additionally, it was also observed that the antifungal activity of fluconazole is considerably reduced under hypoxic conditions. We speculate that the molecular mechanism behind this observation might include Upc2, considering its dual role in activating hypoxia-induced β-glucan masking [97] and conferring azole antifungal resistance [100]. In contrast to *C*. *albicans*, *C*. *tropicalis* is not able to induce β-glucan masking in response to hypoxia, and this species is more susceptible to PMN attack [88]. This is in agreement with the fact that *C*. *tropicalis* mainly infects neutropenic patients [101]. The molecular mechanisms allowing hypoxic adaptation are not completely defined. Nevertheless, it is clear that some *Candida* species take advantage of low-oxygen environments, either generated during infection or imposed by the specific host niche, to thrive by avoiding immune surveillance and escaping from antifungal therapy.

## *Candida* adaptation to temperature shifts is essential for full virulence

The human body temperature is considered to be a potent nonspecific defense against fungal infection, especially in febrile patients, because high temperatures considerably restrict fungal growth [102,103]. The human host presents fever as one of the first responses against a *Candida* infection, thereby exposing the fungal cells to temperatures ranging from 37 °C to 42 °C. These temperature fluctuations profoundly influence many physiological aspects of *C*. *albicans*, including morphology, mating, phenotypic switching, and drug resistance [104].

**Fig 5:**
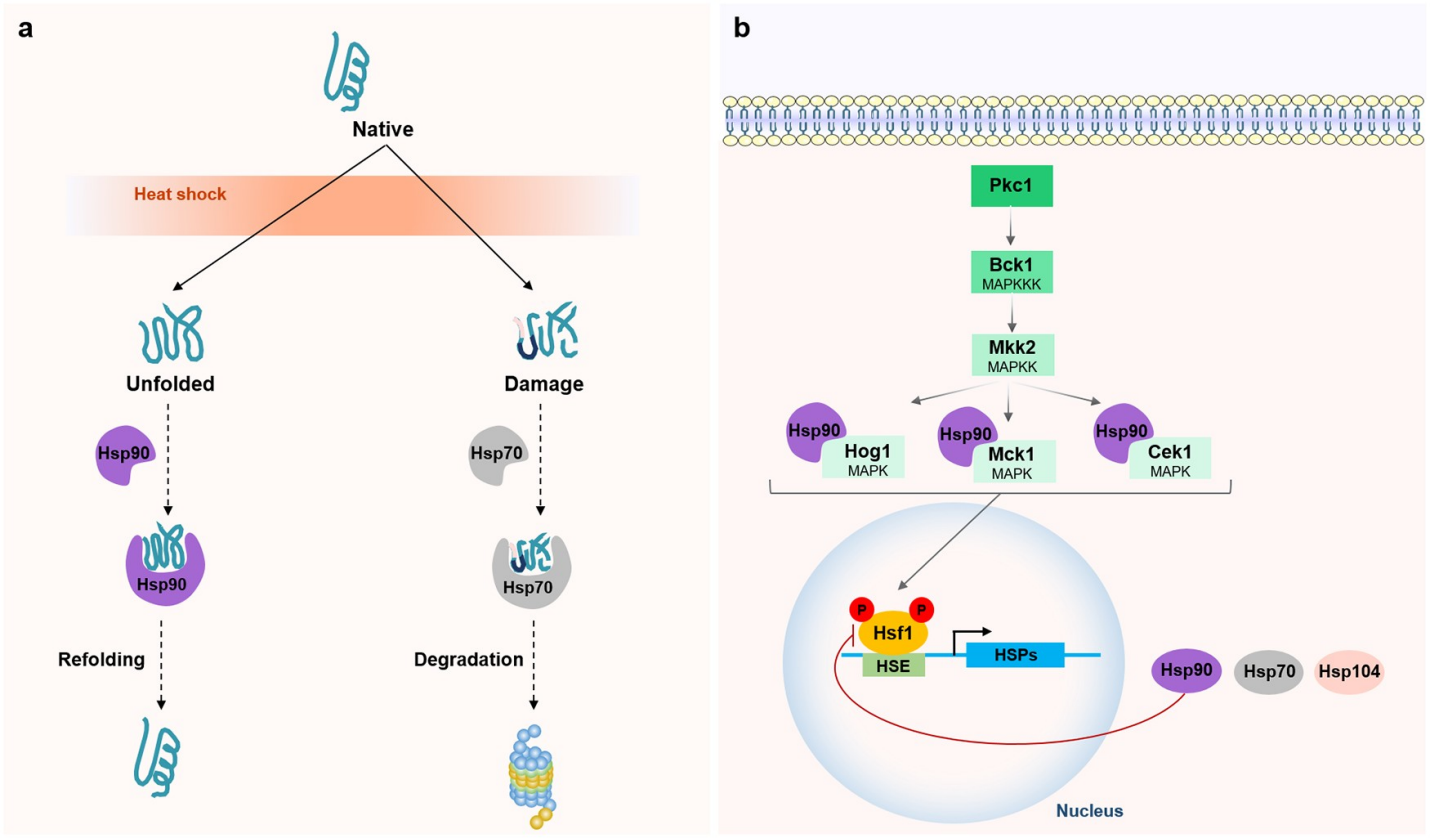
Molecular circuits required for thermal adaptation in *C*. *albicans*. (a) HSPs rescue proteins from unfolding or target damaged proteins for degradation. (b) In response to temperature upshifts, Hsf1 becomes phosphorylated, inducing the expression of HSP genes. After thermal adaptation, Hsf1 returns to basal levels through a negative feedback loop dependent on Hsp90. Long-term adaptation is controlled by Hsp90 through Hog1, Mkc1, and Cek1. HSE, heat shock element; Hsf1, heat shock transcription factor 1; HSP, heat shock protein; MAPK, mitogen-activated protein kinase; MAPKK/MAPKKK, MAPK kinase/ MAPKK kinase.

Changes in ambient temperature are sensed by a broad diversity of mechanisms. One of the most studied pathways is the evolutionarily conserved heat shock response, which mediates thermal homeostasis by controlling the levels of heat shock proteins (HSPs) [105]. HSPs are molecular chaperones sequestered in response to heat shock, rescuing proteins from unfolding or targeting damaged proteins for degradation. In *C*. *albicans*, the expression of HSP genes is activated by the heat shock transcription factor 1 (Hsf1), which becomes phosphorylated in response to temperature elevations, including thermal transitions that mimic fever [106,107]. After adaptation to the exposed temperature, Hsf1 phosphorylation returns to basal levels and several lines of evidence have suggested the existence of a negative feedback loop, in which Hsp90 negatively regulates Hsf1 [107–109]. Besides Hsf1, Hsp90 also controls the activation of other regulators that mediate long-term thermal adaptation (Fig 5). These include several mitogen-activated protein kinase (MAPK) signaling pathways, particularly the Hog1, Mkc1, and Cek1 pathways, which are intimately associated with cell-wall remodeling [110,111]. Other small HSPs such as Hsp12 and Hsp21 have also been identified as crucial for *C*. *albicans* to resist thermal stress [112,113]. HSPs and their associated signaling pathways have been widely implicated in antifungal resistance, emerging as potential antifungal targets to treat *Candida* infections [114]. Moreover, the activation of the Hsf1 transcriptional program in *C*. *albicans* has been associated with increased host cell adhesion, damage, and virulence, reinforcing the importance of this regulon in thermal homeostasis [115,116].

## *Candida* and host microbiota: Avoiding antagonistic interactions in health and disease

The structure of human microbiota is dynamic, often defined by host and environmental factors and also by physical and metabolic interactions between species. While some of these interactions are cooperative, others are antagonistic, and the latter may represent a major obstacle for *Candida*. This concept gained experimental support through studies involving the depletion of commensal microbiota by continued use of broad-spectrum antibiotics, which resulted in *Candida* overgrowth [117,118]. This suggests that some commensal microbial colonizers antagonize *Candida* spp. (and other exogenous pathogens) in order to maintain a homeostatic balance in the host. Some of these interactions are driven by metabolic competition, while others are mediated by quorum-sensing molecules that influence fungal cell behavior and regulate important virulence traits. Although quorum-sensing systems have been explored in great detail for pathogenic bacteria, they are relatively poorly understood in fungi [119]. The *C*. *albicans* molecule farnesol was the first quorum-sensing compound to be identified in an eukaryote [120] and has been the object of intense research. Yet, its precise mode of action remains unclear.

*Lactobacillus* species and *C*. *albicans* are a well-documented example of infectious antagonism [121–123]. *Lactobacilli* are a dominant species of the microbiota of the gastrointestinal and urogenital tracts, and they actively reduce the amount of fungal microbes by producing many fungicidal compounds [121–123]. Other commensal bacteria such as *Bacteroides thetaiotamicron* or *Blautia producta* can antagonize *C*. *albicans* by stimulating intestinal cells to produce antimicrobial peptides [124]. The pathogenic bacterium *Acinetobacter baumanii* has been also reported to interact antagonistically with *C*. *albicans* by binding to hyphae to promote apoptosis [125]. The elucidation of these types of interaction is of particular interest in the quest for novel targets for antifungal therapy, as the inhibitory secreted factors produced by these antagonists appear to have high fungicidal activity.

The disruption of commensal interactions through alterations in immune competence, by changes in environmental host conditions, or via antibiotic therapy may favor the outgrowth and overrepresentation of pathogenic microbes, with these growing at the expense of those organisms that fail to adapt. While antagonist interactions might lower the risk of infection, synergistic interactions during dysbiotic states are associated with increased pathogenesis because microbes can also interact to enhance colonization and persistence. An illustrative example is the infectious synergism established between several *Candida* species (including *C*. *albicans*, *C*. *dubliniensis*, *C*. *tropicalis*, and *C*. *krusei*) and the gram-positive bacterium *Staphylococcus aureus* [126,127]. *Candida* not only provides a substratum for the attachment and colonization of *S*. *aureus* but also facilitates its invasion across mucosal barriers, thereby promoting persistence and systemic infection [128].

## Host immune defenses: How *Candida* species counteract the immune response

Microbial pathogens are constantly surveyed by the innate immune system. Phagocytic cells such as dendritic cells, macrophages, monocytes, and neutrophils play important roles in clearing fungal pathogens from the bloodstream and tissues. Loss of innate immune cells or defects in their antifungal activities have major implications for the host. *Candida* cells are recognized through key PAMPs, some of which are located in the cell wall; for example, β-glucans, chitin, and mannans. These components are sensed by the multiple pattern-recognition receptors (PRRs) expressed by phagocytic cells or secreted (for example, complement components). PPRs mediate binding of the pathogen to the phagocyte, and the PAMP–PRR interactions trigger intracellular signaling pathways within the immune cells that can induce phagocytosis and the production of proinflammatory cytokines and chemokines. In order to attenuate recognition and escape phagocytosis, *Candida* cells are able to actively mask cell-wall PAMPs [129] and secrete specific proteases that target complement opsonization [130]. Alternatively, some *Candida* species can induce their phagocytic uptake into endothelial and epithelial cells and use these cells as “safe houses” by preventing maturation of the phagolysosome and subsequent killing [131]. If none of these strategies is employed, *Candida* cells are likely to be internalized and subjected to a combination of toxic oxidative and nonoxidative mechanisms that attempt to kill an intra- or extracellular yeast cell. These oxidative mechanisms include the production of reactive oxygen and nitrogen species (ROS and RNS, respectively), while nonoxidative killing mechanisms include the release of antimicrobial peptides and the induction of processes related to micronutrient restriction. Of note, while *C*. *albicans* is sensitive to the combinatorial stresses imposed by phagocytes [132], *C*. *glabrata* has adapted to survive within the inhospitable environment of the phagosome. This pathogen mounts robust stress responses against the ROS implemented by the phagocytic cell and neutralizes the phagocytic environment, thereby escaping phagocytosis [133].

### Oxidative, nitrosative, and osmotic/cationic stresses

Phagocytic cells attempt to kill pathogens in part by employing toxic ROS and RNS, either intracellularly or extracellularly, as a major antimicrobial defense mechanism. ROS are produced by the NADPH oxidase complex, a process known as respiratory burst, and include chemicals such as the superoxide anion (O_2_^•^), hydrogen peroxide (H_2_O_2_), and the hydroxyl radicle (^•^OH). Furthermore, ROS production in response to *C*. *albicans* infection has been shown to lead to the recruitment of additional phagocytes, creating a toxic oxidative environment for the fungus [134]. Inside phagocytes, ROS can interact with nitric oxide (NO), generating toxic products such as peroxynitrite [135]. These toxic chemicals cause irreversible damage to the pathogen by interacting with proteins, lipids, and nucleic acids.

**Fig 6:**
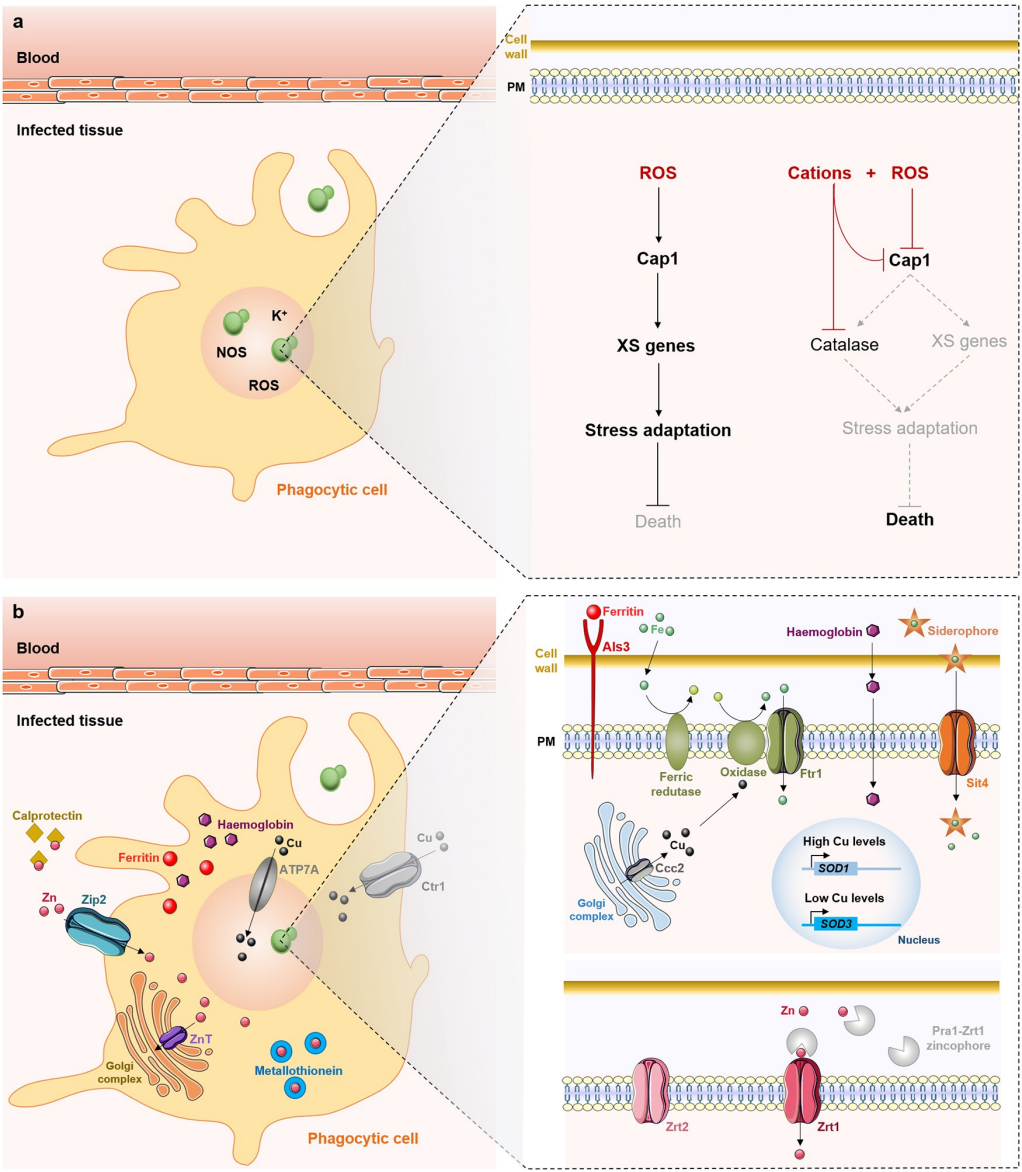
Host immune defenses and adaptation mechanisms displayed by *C*. *albicans* and *C*. *glabrata*. (a) Cap1 plays a key role in the activation of responses to ROS generated by phagocytic cells, leading to the induction of oxidative stress genes (XS genes), including catalase, superoxide dismutases, glutathione peroxidases, and thioredoxins, among others. However, cations inhibit catalase and Cap1, thereby delaying the induction of the oxidative stress response and leading to the death of *C*. *albicans* cells. (b) Host-enforced micronutrient restriction results in reduced iron, copper, and zinc availability, but *C*. *albicans* responds by up-regulating efficient metal-scavenging strategies. Host phagocytes also exploit the toxicity of copper and zinc by pumping these metals in excess into phagosomes to intoxicate internalized pathogens. NOS, nitric oxide species; PM, plasma membrane; ROS, reactive oxygen species; Sod, superoxide dismutase; XS, oxidative stress.

*Candida* species attempt to counteract these stresses by activating cellular responses that include the activation of genes encoding proteins involved in stress detoxification and reparation. These include catalase, superoxide dismutases, glutathione peroxidases, and thioredoxins (Fig 6a) [136–138]. In *C*. *albicans* and *C*. *glabrata*, these stress pathways are regulated largely by the Hog1 stress-activated protein kinase [136,139], the transcription factor Cap1 [140–142], and the Rad53 DNA damage checkpoint kinase [143]. Together with the transcription factor Cta4, these signaling pathways play key roles in orchestrating the responses to osmotic, oxidative, and nitrosative stresses in these species [144]. In this way, these regulators promote the fitness of *C*. *albicans* during systemic infection. Indeed, mutants lacking these genes display attenuated virulence in mice, as well as impaired tolerance to these stresses in vitro and phagocytic survival [145,146]. Curiously, the oxidative stress response is delayed if the fungus is simultaneously exposed to cationic and oxidative stress [147]. This is thought to contribute to the ability of phagocytic cells to efficiently kill invading pathogens (Fig 6a) [132]. Given the importance of these stress response pathways for *Candida* survival, key molecular players involved may represent attractive targets for antifungal development.

### Host-enforced micronutrient restriction

The limitation of micronutrients such as iron, copper, zinc, or manganese is an effective way of controlling the outgrowth of invading microbes. These micronutrients are essential for the survival of both host and pathogen because they function as cofactors for enzymes, transcription factors, and other proteins that play crucial biochemical and cellular functions. However, our immune system attempts to restrict microbial access to these essential elements via a mechanism known as nutritional immunity [148].

Iron has well-studied implications for *Candida* pathogenesis, being a crucial micronutrient for *Candida* growth, survival, and virulence [149]. During systemic candidiasis, the host restricts this metal by increasing the levels of iron-binding proteins, such as ferritin and hemoglobin alpha, and accumulating heme oxygenase (Fig 6b) [150,151]. Both *C*. *albicans* and *C*. *glabrata* have developed efficient iron-scavenging strategies that can overcome these host mechanisms. This contributes to their ability to survive phagocytosis and replicate inside macrophages by using their intracellular storages of iron [152,153]. *C*. *albicans* and *C*. *glabrata* cells exploit sophisticated iron-uptake systems to acquire either free iron [154,155] or iron from host iron-binding proteins, including hemoglobin [156], ferritin [82], and transferrin (Fig 6b). Additionally, the utilization of siderophores promotes resistance to macrophage killing: in *C*. *glabrata*, the Sit1 siderophore-iron transporter mediates iron acquisition, being critical for the survival of the yeast inside macrophages [152].

Copper is also involved in *Candida* virulence, both positively and negatively. The fungal reductive iron-uptake pathway includes multicopper oxidases, and hence, iron acquisition and mobilization depends on copper availability [157]. Interestingly, the host also uses copper as a defense mechanism against *Candida* by pumping excess quantities of this metal into *Candida*-containing phagosomes (Fig 6b) [158]. However, *C*. *albicans* adapts to this potential killing mechanism by differentially modulating the expression of copper- and manganese-dependent SODs (Sod1 and Sod3, respectively) [159]. Sod1 is expressed when copper is in excess, but when copper levels decline, Sod3 is then expressed (Fig 6b) [159]. Thus, during infection, *C*. *albicans* is able to adjust copper uptake and management by using it as an enzymatic cofactor for SOD enzymes [159].

Zinc is an abundant micronutrient that has crucial roles in cellular functionality for both host and pathogen. The host attempts to limit zinc availability for the fungus by depleting extracellular zinc levels, mainly via calprotectin, an antimicrobial peptide expressed by neutrophils that binds zinc and manganese with high affinity (Fig 6b) [160]. Calprotectin promotes the antimicrobial activity of neutrophil extracellular traps (NETs), which are released by neutrophils after sensing large microbes such as *C*. *albicans* hyphae [161–163]. Zinc depletion also occurs inside immune cells as an antifungal mechanism to kill intracellular pathogens such as *C*. *albicans* and *C*. *glabrata* [164]. During infection, macrophages deplete intracellular zinc by pumping it into the Golgi apparatus via specific ZnT-type zinc transporters (Fig 6b) and increasing the expression of zinc-binding metallothioneins [165]. Additionally, macrophages up-regulate the zinc importer *ZIP2* to increase the intracellular levels of zinc (Fig 6b) [166]. This combination of strategies depletes zinc from the extracellular environment while dealing with the increased metabolic demands associated with microbial clearing [166]. To overcome zinc depletion, *C*. *albicans* overexpresses *ZRT1* and *ZRT2* genes, encoding zinc uptake transporter systems Zrt1 and Zrt2 (Fig 6b). Both transporters are regulated by the zinc finger transcription factor Zap1 (also known as Csr1) [167,168] and by pH [79]. Zinc transporters play important roles in *Candida* pathogenesis because overexpression of Zrt2 increases *C*. *albicans* virulence [169]. In addition to functioning as a zinc transporter, Zrt1 also serves as a receptor for the Pra1 zincophore [79,168], a secreted protein that binds and sequesters zinc from host cells during *C*. *albicans* invasion (Fig 6b) [170]. Similarly to copper, zinc has also been reported to be pumped in higher amounts into the phagosome to intoxicate internalized pathogens, constituting an important mechanism of killing (Fig 6b) [171].

## Environment-triggered biofilm formation and antifungal resistance

So far, we have described major molecular circuits required by *Candida* species to counteract several constraints they face in the human host. The ability of *Candida* to adapt to these stresses imparts the flexibility to colonize diverse host niches. The physiological capacity to respond efficiently to stress and survive hostile environments also endows the fungal cells with the advantage of being better prepared for future insults [172,173]. The generation of biofilms might represent another strategy to resist harsh conditions and persist in the human host.

The *Candida* species most frequently associated with the formation of biofilms, either in host tissues or implanted medical devices, are *C*. *albicans*, *C*. *glabrata*, *C*. *tropicalis*, and *C*. *parapsilosis* [174]. Biofilms represent three-dimensional communities of adherent cells, with distinct biological properties, that are embedded in a self-synthesizing extracellular matrix (ECM) composed predominantly of proteins, glycoproteins, carbohydrates, lipids, and nucleic acids [175]. The ECM helps to maintain the overall structural integrity of the biofilm, and it also acts as a physical barrier to drug penetration. Consequently, biofilm cells can survive drug concentrations more than a thousand times higher than those defined for planktonic cells [176]. This phenotype is partly associated with the sequestration of drugs by the biofilm ECM and partly with the occurrence of a subpopulation of so-called “persister cells”. Persister cells exhibit a dormant-like physiology that has been demonstrated to make them highly resistant to antifungals [177]. These features contribute to the intrinsic resistance of *Candida* biofilms to conventional antifungal treatments, the host immune system, and other environmental perturbations, making biofilm-based infections a clinical challenge.

Genome-wide transcriptional profiling and proteomic approaches have identified hundreds of genes that are differentially expressed between *C*. *albicans* biofilm and planktonic cells. The up-regulation of glycolytic and sulfur amino acid genes, similar to what is observed when cells grow under hypoxia, suggests that *Candida* biofilms constitute a heterogeneous environment with hypoxic niches [178]. Moreover, more than 50 transcriptional regulators and 101 other genes have functionally validated roles in the formation of *Candida* biofilms [179–181]. Some of these play important roles in hyphal formation, adhesion, drug resistance, and matrix production (all intrinsic characteristics of biofilms), as well as in stress adaptation. It is not surprising, then, that adaptation to specific environmental niches modulates the ability of cells to form biofilms and, consequently, to resist antifungals [54,55,58,59,182–184].

## Final remarks and future perspectives

*Candida* cells regulate specific sets of genes, including many involved in an array of stresses and metabolic pathways, in order to thrive and persist in the human host. In addition to conferring metabolic flexibility and stress resistance, the physiological reprogramming has been associated with enhanced virulence through impaired immune recognition, increased biofilm formation, and/or acquired antifungal tolerance and resistance. Although remarkable progress has been made in the last few decades in our understanding of the impact of host-derived stresses on *Candida* physiology and pathogenicity, many details remain unclear. During an infection, *Candida* cells are exposed to multiple environmental constraints, sometimes imposed consecutively, and at other times imposed simultaneously. Yet, in vitro experiments are predominantly designed to study individual environmental signals, often at single time points, rather than combinatorial stresses over time. Much progress has been achieved using the first approach. While this has given us valuable insights, it rather oversimplifies biological reality. The analysis of combinatorial stresses and of the dynamism of these inputs would mimic host conditions more closely and reveal more detailed views of which stress or stresses prevail and dictate the outcome of different types of infection. The same principle applies to infection and biofilm models, in which usually interactions between only a few different microbial populations have generally been examined. Most knowledge in the field comes from studies of either *C*. *albicans* or *C*. *glabrata*. Yet, the regulatory circuits required to effectively respond to each constraint, including antifungal treatments, differ considerably between the different *Candida* species, illustrating how heterogeneous these pathogens are. With the unprecedented emergence of multidrug resistant species such as *C*. *auris*, there is an urgent need to develop new effective antifungals. The integration of omics data with in vivo models, which mimic host conditions more closely, is now a powerful strategy to unravel molecular processes underlying adaptive phenotypes. These platforms have already produced novel lines of research and improved the identification of new potential therapeutic targets for vaccine and antifungal drug development, enhancing our ability to develop novel strategies to fight *Candida* infections.
